# Hemispheric Asymmetry in White Matter Connectivity of the Temporoparietal Junction with the Insula and Prefrontal Cortex

**DOI:** 10.1371/journal.pone.0035589

**Published:** 2012-04-19

**Authors:** Aaron Kucyi, Massieh Moayedi, Irit Weissman-Fogel, Mojgan Hodaie, Karen D. Davis

**Affiliations:** 1 Division of Brain, Imaging and Behaviour – Systems Neuroscience, Toronto Western Research Institute, University Health Network, Toronto, Ontario, Canada; 2 Institute of Medical Science, University of Toronto, Toronto, Ontario, Canada; 3 Division of Neurosurgery, Toronto Western Hospital, Toronto, Ontario, Canada; 4 Department of Surgery, University of Toronto, Toronto, Ontario, Canada; Beijing Normal University, Beijing, China

## Abstract

The temporoparietal junction (TPJ) is a key node in the brain's ventral attention network (VAN) that is involved in spatial awareness and detection of salient sensory stimuli, including pain. The anatomical basis of this network's right-lateralized organization is poorly understood. Here we used diffusion-weighted MRI and probabilistic tractography to compare the strength of white matter connections emanating from the right versus left TPJ to target regions in both hemispheres. Symmetry of structural connectivity was evaluated for connections between TPJ and target regions that are key cortical nodes in the right VAN (insula and inferior frontal gyrus) as well as target regions that are involved in salience and/or pain (putamen, cingulate cortex, thalamus). We found a rightward asymmetry in connectivity strength between the TPJ and insula in healthy human subjects who were scanned with two different sets of diffusion-weighted MRI acquisition parameters. This rightward asymmetry in TPJ-insula connectivity was stronger in females than in males. There was also a leftward asymmetry in connectivity strength between the TPJ and inferior frontal gyrus, consistent with previously described lateralization of language pathways. The rightward lateralization of the pathway between the TPJ and insula supports previous findings on the roles of these regions in stimulus-driven attention, sensory awareness, interoception and pain. The findings also have implications for our understanding of acute and chronic pains and stroke-induced spatial hemineglect.

## Introduction

The temporoparietal junction (TPJ) is a key component of a right-lateralized ventral attention network (VAN) that also includes structures such as the anterior insula (aINS), and the inferior frontal gyrus (IFG) [1]. Functional MRI (fMRI) studies have consistently shown that these right-lateralized regions are activated by salient stimuli in visual, auditory and somatosensory modalities including prolonged pain, with a preference for behaviourally-relevant stimuli [2], [3], [4], [5], [6]. Lesions to areas within the VAN and their surrounding white matter are a common neural substrate of left unilateral spatial neglect, suggesting that regions in this right-lateralized network play a specialized role in spatial awareness [7], [8]. Transcranial magnetic stimulation of the right TPJ results in abnormal orienting of stimulus-driven attention [9]. Furthermore, resting state BOLD studies demonstrate that areas within the VAN, particularly the TPJ and aINS/IFG, have correlated intrinsic fluctuations in activity in the right hemisphere [10], [11]. However, despite extensive fMRI, lesion and stimulation studies on the VAN, the anatomical basis of this network's right-lateralized properties remains poorly investigated.

In recent years, diffusion-weighted MRI (DW-MRI) has emerged as an invaluable tool for investigating *in vivo* connectional anatomy in the human brain [12]. In DW-MRI, the signal is sensitized to anisotropic diffusion of water, which occurs in brain white matter and is characterized by greater diffusion along an axon compared to across an axon. If the diffusion profile in each voxel is fit to a tensor model, principal diffusion directions can be estimated and white matter pathways can be traced. This method of deterministic “streamline tractography,” however, is limited in that tracing is poor near gray matter, where anisotropy is low but white matter is still present. Thus alternative techniques such as probabilistic tractography [13], [14] have been developed to improve sensitivity. In probabilistic tractography, a probability distribution representing uncertainty in fiber orientation is modeled at each voxel. A large number (usually thousands) of streamlines are drawn between two points to build up a connectivity distribution, and the number of successful connections is counted. This approach is advantageous because pathway tracing does not stop near gray matter, multiple fiber populations can be modeled [15], and quantitative measures of connection likelihood can be obtained. Despite inherent limitations of probabilistic tractography (reviewed in [16]), the technique is useful especially when *a priori* connections are known.

Anatomical connections between regions of the VAN have been identified in the monkey and human. The arcuate fasciculus (AF) and subcomponent III of the superior longitudinal fasciculus (SLF III) connects the TPJ with the IFG [17], [18], [19], and the extreme capsule connects the TPJ with the insula [20], [21]. DW-MRI studies indicate that temporoparietal regions are also connected with the aINS and pars triangularis of the IFG via the extreme capsule [19], [22]. Recently, right-lateralization of the SLF III was identified, and the degree of SLF II right-lateralization was correlated with performance on tasks involving visuospatial attention [23]. However, it remains unknown whether hemispheric differences exist in the strength of connections between specific VAN gray matter regions.

Therefore, the aim of this study was to determine the strength and laterality of the structural connectivity between the TPJ and regions within the VAN and elsewhere that are involved in salience detection, including pain. We used DW-MRI and probabilistic tractography to characterize and compare white matter connectivity profiles of the right TPJ (rTPJ) and left TPJ (lTPJ) to test the hypothesis that the TPJ exhibits stronger connectivity with the insula, IFG, cingulate cortex, thalamus and putamen in the right compared to left hemisphere.

## Methods

### Subjects and image acquisition

Anatomical data were acquired from 25 healthy, right-handed subjects (14 males, mean age ± SD: 28.3±4.27 years; 11 females, mean age ± SD: 26.9±3.42 years). Informed written consent was obtained from all study participants for procedures approved by the University Health Network Research Ethics Board.

Images were acquired with a 3-Tesla GE MRI system at Toronto Western Hospital fitted with an eight-channel phased-array head coil. For each subject, two different acquisitions were obtained to validate the presence of and lateralization in connectivity: one with 25 and the other with 60 diffusion-encoding directions (b = 1,000 s/mm^2^). For each acquisition, one B0 scan was acquired at the beginning of the run, and the parameters were as follows: repetition time (TR) = 12,000 ms, field of view: 24×24 cm^2^, 128×128 matrix, 1.875×1.875 mm^2^ in-plane resolution, 3 mm thick axial slices. We also obtained high-resolution T1-weighted images using the IR-FSPGR sequence with the following parameters: 160 axial slices, 0.94×0.94×1.0 mm^3^ voxels, 256×256 matrix, field of view = 24×24 cm, flip angle = 20°, TE = 5 ms, TR = 12 ms, TI = 300 ms.

### Diffusion Image Preprocessing

Preprocessing was carried out with Functional MRI of the Brain Software Library (FSL, v.4.1; http://www.fmrib.ox.ac.uk/fsl) [24]. Diffusion-weighted images (DWI) and T1-weighted images were skull-stripped using the Brain Extraction Tool [25]. DWI scans were corrected for motion and eddy currents (Jenkinson et al., 2002). To obtain isotropic voxels, DWI scans were down-sampled to 3×3×3 mm^3^. Probability distributions at each voxel were then calculated for two possible fibre orientations to account for crossing fibres within voxels [13], [15]. Affine registration transformation matrices among diffusion, T1 and standard MNI152 stereotaxic spaces were created using the FMRIB's Linear Image Registration Tool (FLIRT) [26].

### Seed and Target Definition

**Figure 1:**
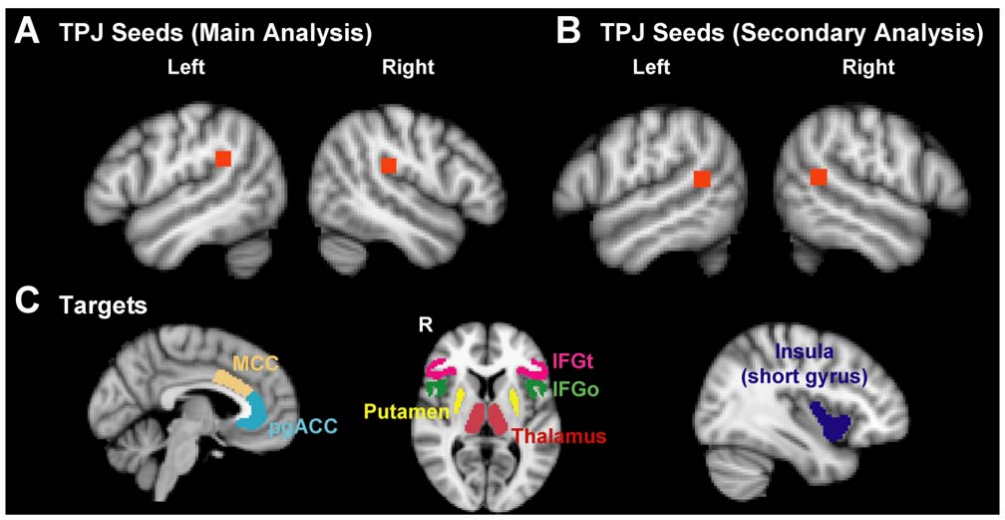
Regions of interest, displayed on the MNI152 stereotaxic brain for A) TPJ seeds where tractography was initiated (top row displays seeds for primary analysis, and bottom row displays seeds for secondary analysis) and B) targets. For targets, the left image displays perigenual anterior cingulate cortex (pgACC) and mid-cingulate cortex (MCC), the middle image displays bilateral pars triangularis of the inferior frontal gyrus (IFGt), pars opercularis of the inferior frontal gyrus (IFGo), putamen and thalamus, and the right image displays the short gyrus of the insula.

Seed regions in the TPJ were manually defined on the standard (MNI 152) template brain (voxel size = 2×2×2 mm) based on TPJ coordinates reported in previous anatomical and functional imaging studies [1], [27]. Anatomically, the TPJ includes the posterior superior temporal sulcus (STS), inferior parietal lobule (IPL) and lateral occipital cortex, whereas functionally the TPJ includes portions of the posterior STS and superior temporal gyrus (STG) as well as the ventral part of the supramarginal gyrus (SMG) (reviewed in [1]). To define the TPJ in a manner that encompasses aspects of both the functional and anatomical definitions, a 5×5×5 voxel mask (volume = 1000 mm^2^) was drawn in gray matter around center of mass coordinates [MNI: x = 50; y = −28; z = 22] for rTPJ and [MNI: x = −50; y = −42; z = 26] for lTPJ. These seed locations (see Figure 1A) were similar in both hemispheres, encompassing Brodmann areas 40 and 22, including posterior parts of the STG and ventral parts of the SMG and IPL, and overlapping with regions within the TPJ that are activated in multimodal studies of attention [3], [4], [5]. We used TPJ seeds with different y and z coordinates in the two hemispheres rather than mirror images to reflect underlying asymmetries in sulcal anatomy. A more anterior R TPJ compared to L TPJ is concordant with the relative locations of activations in these regions in our previous fMRI studies of attention [4], [5].

While the main results are presented for the seed regions based on TPJ coordinates described above (for both 23- and 60-direction data), a secondary analysis was performed (only for the 60 direction acquisition) with different seed locations within the TPJ to verify that the findings were relevant to a broader distribution than the initially defined TPJ location. These secondary TPJ seeds were 5×5×5 voxel masks drawn in gray matter around center of mass coordinates [MNI: x = 54; y = −42; z = 16] for rTPJ and [MNI: x = −54; y = −50; z = 14] for lTPJ (Figure 1B). To define these regions, the MNI Structural Atlas [28] was used to display the gray matter of the parietal and temporal lobes thresholded at *p*>0.5 (i.e., greater than 50% of the population from which the atlas was derived) in both hemispheres, and the TPJ was drawn at the point where the two lobes meet. The resulting seed location was in line with the area of the TPJ that is considered to be part of the VAN [10] and that is consistently activated in studies of stimulus-driven attention [27]. The TPJ locations for both the main analysis and the secondary analysis are consistent with the area within the TPJ that has been classified as “anterior TPJ” by connectivity-based parcellation [29].

Fourteen target regions (including homologous regions in both hemispheres) were defined bilaterally (Figure 1C). These targets included the insula, IFG pars opercularis (IFGo), IFG pars triangularis (IFGt), anterior cingulate (ACC), mid-cingulate (MCC), putamen and thalamus. The insula and IFG locations were based on previously reported right-lateralized functional co-activation with the TPJ in studies of stimulus-driven attention and salience detection [1], [3], [4], [5]. The cingulate and thalamus targets were included because they often co-activate with the TPJ [2], [3], [4], [5], [6], but in a non-lateralized fashion. The putamen was included because it shows the same response profile as VAN regions during prolonged pain [2], but asymmetric connectivity was not expected.

Subcortical target regions were defined using the Harvard-Oxford subcortical probabilistic atlas (http://www.cma.mgh.harvard.edu/fsl_atlas.html) with the probability volumes of the putamen and thalamus thresholded at *p*>0.9 so as to exclude neighbouring gray and white matter. Cortical target regions were defined using Freesurfer's (http://surfer.nmr.mgh.harvard.edu) automated gray matter parcellation (with the aparc2005 atlas) [30] and were transformed to MNI 152 standard space. The IFG was divided into the IFGo and IFGt as these subregions have different cytoarchitecture and different white matter connectivity profiles [19], [31], [32]. The insula ROI encompassed the short gyrus, which includes regions that are classified as anterior and middle portions of the insula [33], [34], [35]. The more posterior portions of the insula were excluded from the analysis because the close proximity to the TPJ may preclude accurate tractography. Two subregions of the cingulate cortex were selected, the MCC and pregenual ACC (pgACC), because they exhibit different cytoarchitecture, anatomical connectivity, and functionality [36]. Prior to probabilistic tractography, all defined seeds and targets were converted from standard to individual subject space.

### Probabilistic Tractography

For each TPJ seed in each subject, 5000 streamline samples in each seed voxel were drawn on principal diffusion directions. Connectivity was assessed between each TPJ seed and all ipsilateral and contralateral targets. Given that with probabilistic tractography long connections inherently result in lower probability values than short connections, we applied a distance normalization algorithm that multiplies the number of samples (out of 5000) from a seed voxel reaching the target by the average distance to the target, as implemented in FSL. This effectively gives greater weighting to longer connections [37], [38] to ensure that hemispheric asymmetries are less likely to occur because of hemispheric differences in pathway length between homologous connections. This weighting also allowed a normalized comparison of connectivity for target regions located at different distances from the TPJ seeds. We also used probabilistic tractography values without distance normalization to provide a sense of the detectability of each connection and to guide our analysis (Table 1). If a connection had an average non-distance-normalized connectivity value of <2.0 (i.e. <0.04% of the 5000 samples from seed reaching target) or a success rate of <50% across subjects, the connection was excluded from statistical analyses (described below) to avoid analysis of potential false positives.

**Table 1:** Success rate (percentage of subjects that had a connection value >2.0 out of 5000 samples), mean and standard error of connectivity values with the TPJ for each ipsilateral target (non-distance-corrected).

Ipsilateral Target		25 directions		60 directions	
		lTPJ	rTPJ	lTPJ	rTPJ
Putamen	Success rate	84%	84%	84%	92%
	Mean	16.28	16.13	21.21	13.47
	Standard error	3.93	6.36	7.77	3.71
Thalamus	Success rate	92%	80%	92%	92%
	Mean	31.15	26.4	54.53	47.22
	Standard error	11.5	9.37	17.72	25.41
pgACC	Success rate	0	0	24%	20%
	Mean	1.15	1.16	1.96	1.5
	Standard error	0.057	0.052	0.36	0.17
MCC	Success rate	4%	4%	8%	16%
	Mean	1.24	1.26	1.31	1.55
	Standard error	0.12	0.082	0.09	0.15
Insula	Success rate	96%	100%	100%	100%
	Mean	30.18	90.81	32.16	73.45
	Standard error	11.89	30.83	11.63	13.45
IFGo	Success rate	100%	96%	100%	100%
	Mean	122.48	29.49	90.35	31.99
	Standard error	30.85	10.18	29.36	11.27
IFGt	Success rate	100%	96%	100%	88%
	Mean	61.87	20.61	52.28	15.55
	Standard error	18.48	6.65	25.93	5.33

Abbreviations: rTPJ = right temporoparietal junction, lTPJ = left temporoparietal junction, pgACC = pregenual anterior cingulate cortex, MCC = mid-cingulate cortex, IFGt = inferior frontal gyrus (pars triangularis), IFGo = inferior frontal gyrus (pars opercularis).

### Statistical Analysis

At the individual subject level, the distance-normalized probabilistic tractography output values were averaged for non-zero voxels within the TPJ seed for each connection that was examined. Across subjects, these mean values were averaged for each seed-target pair. Since differences in size of the 13 targets could lead to higher connectivity values based on greater target size alone, unrelated to connection strength or density, we divided the distance-normalized value by target size then rescaled by multiplying by the mean of all target sizes (as in [38]). Group-level statistical analyses were carried out on the resulting values, which we refer to as “connection strength.”

A lateralization index [39], [40], [41] was calculated on an individual subject basis with connection strength values as follows:
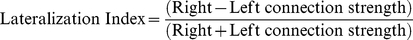
Thus, positive values indicate a right lateralization and negative values indicate a left lateralization. Subjects who did not have any connections between a given seed-target pair were excluded from the lateralization index calculation for that pair (<0.01% of all tested connections across subjects). A one-sample *t*-test (Bonferroni-corrected) was conducted on the lateralization index values for each connection to identify asymmetry inferred from within-individual differences in connectivity between hemispheres (as in [41]). To test for sex differences (using the 60 direction acquisition), we ran a repeated-measures ANOVA with lateralization index values entered for each target as a within-subjects factor and with sex entered as a between-subjects factor. *Post-hoc* independent samples *t*-tests (p<0.05, Bonferroni-corrected) were used to compare lateralization index values between the sexes for each target.

## Results

**Figure 2:**
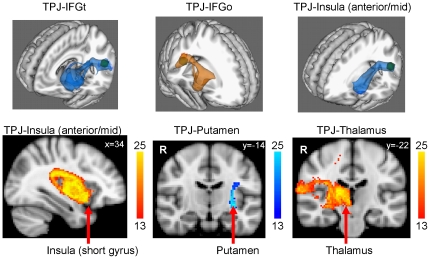
Examples of connections between the TPJ and specified targets, displayed in 3D (top row) and 2D (bottom row) on the MNI152 stereotaxic brain. All statistical maps display voxels that are positive for a given connection in >50% of subjects (colour maps in 2D images represent the number of subjects contributing to a voxel). The TPJ seed is shown as a green cube in the 3D images. Right hemisphere connections are shown in red/yellow/orange and left connections are shown in blue. Abbreviations: IFGt = inferior frontal gyrus (pars triangularis), IFGo = inferior frontal gyrus (pars opercularis).

Connections were identified between both TPJ seeds and all ipsilateral targets for each DWI acquisition. In Figure 2, examples of connections are displayed in 3D to show the courses of identified tracts, and 2D connections are displayed to show examples of across-group statistical maps on selected brain slices. There was weak or absent TPJ connectivity with the pgACC and MCC within each hemisphere, and these connections had average non-distance-normalized connection values of <2.0 in each acquisition for subjects who exhibited a connection (Table 1).

All contralateral connections had average non-distance-normalized connection values <2.0 or success rates of <50% across subjects (Table 2). Thus connections between the TPJ and contralateral regions as well as ipsilateral cingulate regions were excluded from further statistical analysis. All other seed-target pairs exhibited connections that traversed similar pathways within each hemisphere, suggesting that these connections were homologous. For the ipsilateral targets that were included in our statistical analysis.

**Table 2:** Success rate (percentage of subjects that had a connection value >2.0 out of 5000 samples), mean and standard error of connectivity values with the TPJ for each contralateral target (non-distance-corrected).

Contralateral Target		25 directions		60 directions	
		lTPJ	rTPJ	lTPJ	rTPJ
Putamen	Success rate	4%	16%	48%	36%
	Mean	1.27	2.14	2.14	2.35
	Standard error	0.07	0.39	0.26	0.39
Thalamus	Success rate	44%	36%	48%	52%
	Mean	3.25	3.27	7.8	4.22
	Standard error	0.58	0.85	2.51	0.94
pgACC	Success rate	0	0	24%	4%
	Mean	1.14	1.09	1.56	1.26
	Standard error	0.04	0.03	0.16	0.06
MCC	Success rate	0	0	8%	0
	Mean	1.04	1.17	1.21	1.19
	Standard error	0.02	0.05	0.07	0.05
Insula	Success rate	20%	20%	40%	24%
	Mean	1.56	1.73	2.24	2.06
	Standard error	0.17	0.19	0.33	0.35
IFGo	Success rate	0	12%	4%	12%
	Mean	1.07	1.39	1.16	1.4
	Standard error	0.03	0.14	0.05	0.1
IFGt	Success rate	8%	8%	4%	0
	Mean	1.22	1.34	1.37	1.18
	Standard error	0.12	0.11	0.09	0.05

Abbreviations: rTPJ = right temporoparietal junction, lTPJ = left temporoparietal junction, pgACC = pregenual anterior cingulate cortex, MCC = mid-cingulate cortex, IFGt = inferior frontal gyrus (pars triangularis), IFGo = inferior frontal gyrus (pars opercularis).

**Figure 3:**
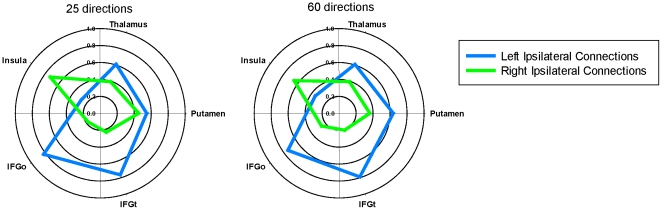
Polar plots displaying proportion of right versus left target connections with the ipsilateral TPJ. Values are based on distance-normalized, target size-normalized connection strengths averaged across data a given DWI acquisition. Abbreviations: Put = putamen, IFGt = inferior frontal gyrus (pars triangularis), IFGo = inferior frontal gyrus (pars opercularis).

**Figure 4:**
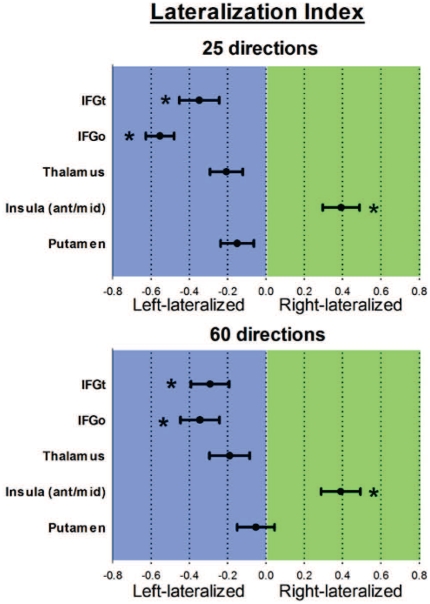
Lateralization index values for each target's connectivity with the TPJ. One sample t-tests revealed significant right lateralization of TPJ-insula connectivity as well as left lateralization of TPJ-IFGo and TPJ-IFGpt connectivity using both DWI acquisitions (*p*<0.05, Bonferroni-corrected). Abbreviations: IFGt = inferior frontal gyrus (pars triangularis), IFGo = inferior frontal gyrus (pars opercularis).

Figure 3 depicts the proportion of right versus left mean connection strength values. These polar plots illustrate the predominance of TPJ connections with the right versus left insula, and also the left predominance of TPJ connections with the IFG with both MRI acquisitions.The lateralization index was used to quantify these findings. The average lateralization index values are shown in Figure 4. One-sample *t*-tests revealed significant rightward lateralization for TPJ-insula connectivity for the 25 and 60 direction acquisitions (*p*<0.05, Bonferroni-corrected). Significant leftward lateralization was found for TPJ-IFGo and TPJ-IFGt connections for both acquisitions. No lateralization was found for TPJ-putamen or TPJ-thalamus connections.

The secondary TPJ seed location (Figure 1B) in the 60-direction acquisition yielded a similar pattern of results as described above. Specifically, a significant rightward lateralization was found for TPJ-insula connectivity (average lateralization index = 0.40) and significant leftward lateralization was found for TPJ-IFGo connectivity (average lateralization index = −0.46), but no lateralization was found for IFGt, thalamus or putamen.

A repeated-measures ANOVA on data from the 60-direction acquisition revealed a significant main effect of target (*F* = 26.557, *p*<0.01) and a significant target by sex interaction effect (*F* = 5.627, *p* = 0.002) for lateralization index values corresponding to the main TPJ seed analysis. *Post-hoc t*-tests revealed that TPJ-insula connectivity was more strongly right-lateralized in females (*p*<0.05, Bonferroni-corrected), however there were no significant sex differences in TPJ connectivity with any other target regions.

## Discussion

This study provides evidence that the human TPJ is strongly structurally connected to regions implicated in attention, salience detection and pain, including the anterior/mid insula, IFG, thalamus and putamen. Furthermore, the TPJ-insula connectivity was strongly right-lateralized whereas the TPJ-IFG connectivity was strongly left-lateralized. Overall, the lateralized TPJ-insula findings were consistent across different DWI acquisitions, and complement functional imaging, lesion, and brain stimulation studies showing these areas to be key nodes in the right-lateralized VAN (reviewed by [1]). Therefore, our findings provide an anatomical basis for the right-lateralized TPJ-aINS functional co-activation and resting state functional connectivity previously reported with functional MRI [5], [10].

### TPJ-Insula Connectivity

Structural connections between the TPJ and the insula have previously been identified in the primate [20], [21] and human [19], [22] brains. In the left hemisphere, a ventral pathway identified with DW-MRI between the posterior middle temporal gyrus (an area adjacent to the TPJ) and aINS was anatomically attributed to the middle longitudinal fascicle (MdLF) and extreme capsule [22], consistent with connections shown with autoradiographic tracing techniques [18], [21]. A dorsal pathway, consisting of the SLF III and the AF, has also been shown to connect the SMG as well as the STG with the aINS in the right hemisphere [19]. The SLF III has previously been shown to be right-lateralized in right-handed individuals [23], consistent with our findings. In our study, dorsal and ventral pathways between TPJ and insula were identified in both hemispheres. We suggest that dorsal contributions originate from parietal as well as STG portions of the TPJ seed and traverse through the AF/SLF, whereas ventral contributions originate mainly from portions of the STG traverse through the MdLF and extreme capsule.

Interestingly, the finding of rightward asymmetry in TPJ-insula connectivity may have implications for the basis of spatial hemi-neglect, a neurological condition in which the patient exhibits deficits in attention to and awareness of one side of space. Neglect typically occurs following lesions in the right hemisphere and results in left-sided attention deficits, but rarely occurs after left hemisphere damage [8], [42]. While lesions in white matter surrounding the rTPJ are known to produce neglect, increasing evidence implicates white matter disconnection between the frontal and parietal lobes in neglect [43]. A recent study comparing large-scale white matter networks of the right versus left hemisphere found that temporoparietal areas (SMG and angular gyrus) exhibited rightward asymmetry in “betweenness centrality,” a measure of the extent to which a region acts as a bridge between connected node pairs [44]. This suggests that TPJ-insula connectivity may not be the only pathway connected with the TPJ that exhibits rightward asymmetry. However, it is possible that disruptions in the structural connectivity between the TPJ and aINS specific to the right hemisphere play a role in generating some symptoms of neglect. Patients with neglect exhibit disrupted functional connectivity of the right aINS with regions within the VAN [11]. In line with the notion that the aINS represents all subjective feelings of the body [45], disrupted connectivity with the aINS could, for example, be an anatomical correlate of “personal neglect” in which the patient loses awareness of the contralesional half of their own body [46]. Recent DW-MRI studies have provided insights into the major association fibre tracts involved in neglect [47], [48], [49]. Future investigations of potential disruptions in TPJ-insula structural connectivity could build upon these studies.

A rightward asymmetry in TPJ-insula connectivity has important implications pertaining to acute and chronic pains. The prolonged salience of acute pain is reflected by sustained activation of the rTPJ [2]. Furthermore, task and resting state functional MRI, as well as gray matter studies of chronic pain consistently implicate the anterior/mid-insula [50], [51], [52], [53], [54]. These abnormalities could arise from or induce aberrant white matter connectivity of the insula [55]. Thus, it is possible that disruption of the prominent TPJ-insula pathway in the right hemisphere affects the salience system in persons with chronic pain. The finding of greater TPJ-insula rightward asymmetry in females compared to males is novel, but difficult to explain since studies on the role of the TPJ in attention generally do not investigate sex differences, although many chronic pain disorders are female dominant.

### TPJ-IFG Connectivity

The IFGo and IFGt express distinct connectivity patterns [31]. With respect to their connectivity with the temporoparietal area, Umarova et al. [19] suggested that the right IFGt is more likely to be connected via a ventral pathway through the extreme capsule, whereas the IFGo is more likely to be connected via a dorsal pathway through the AF/SLF. Our data suggest that in both hemispheres, the IFGt and IFGo are connected to the TPJ via both dorsal and ventral pathways. Discrepancies between our results and previous findings are likely due to differences in locations of regions of interest where tracking was initiated and terminated.

Contrary to our initial hypothesis, we found leftward (not rightward) asymmetry connecting the TPJ with IFGo and IFGt. These findings are not surprising given that the left IFG is Broca's area in right-handed subjects, a key component of the human language system. It is well established that structural connectivity via the AF between Broca's area and language-related regions of the temporal lobe (Wernicke's area) is lateralized to the left hemisphere in right-handed individuals [39], [40], [56]. It is likely that the temporal regions of the TPJ that we tracked from overlapped with Wernicke's area, giving rise to leftward asymmetry in IFG connectivity. This extends the hypothesis that the left language network and right VAN are homologous [10]. However, a pure dichotomy of hemispheric function is unlikely, as white matter connectivity between the TPJ and IFG in the right hemisphere has been associated with behavioural performance in grammar learning [57]. The right IFG is also implicated in pain processing, anticipation and modulation, and abnormal gray matter volume has been identified in this region in chronic pain [58], [59], [60], [61]. Thus a disruption in the connection between the right TPJ and IFG may have a role in chronic pain.

### Putamen and Thalamus

The putamen and thalamus are anatomically connected with the temporoparietal area in non-human primates [21], consistent with connections we identified. Both of these regions are tonically activated during painful but not non-painful stimulation, in a similar manner to VAN regions [2]. Lesions to the putamen result in reduced pain sensitivity and pain-related brain activation [62]. The potential role of TPJ-putamen connectivity in this disrupted processing is likely complex, as the putamen is also anatomically connected to a number of other regions involved in pain, including the insula, ACC and thalamus [62]. Since no laterality was found for putamen or thalamus connections with the TPJ, the relationship of these target regions with the VAN remains unclear.

### Cingulate

The tractography methodology in this study was not able to clearly identify white matter connectivity between the TPJ and pgACC/MCC. Although the TPJ and pgACC/MCC often co-activate with one another during stimulus-driven attention and pain, these activations are not always lateralized to the right hemisphere [2], [3], [63], [64]. The ACC is anatomically connected with the insula in non-human primates [65], [66]. Furthermore, the pgACC/MCC and aINS often co-activate in perceptual and cognitive experiments, even in absence of VAN engagement [67], and show abnormal activation to cognitive and emotional tasks in chronic pain [54]. The aINS and pgACC/MCC also exhibit intrinsic functional connectivity in the “salience network” [35], [68], that has stronger connectivity in the right hemisphere [69] and is disrupted in chronic pain [53]. It remains an open question whether structural connectivity between the aINS and pgACC/MCC is stronger in the right hemisphere and whether connectivity in the salience network is functionally and anatomically related to rightward asymmetry in TPJ-insula connectivity.

### Anatomical versus Functional Connectivity

Emerging evidence from the field of human “connectomics” [70] indicates that white matter anatomical connectivity is intricately related to functional co-activation and intrinsic functional connectivity. Functional connectivity refers to highly synchronous low frequency oscillations between brain areas [71] and may reflect actual anatomical connectivity or merely common inputs to the brain areas. Areas that commonly co-activate with one another during stimulus or task conditions are likely to exhibit intrinsic functional [72] as well as structural [38] connectivity. Studies comparing structural with resting-state functional connectivity throughout the brain suggest that white matter connectional strength is largely predictive of the degree of functional connectivity between gray matter regions, but functional connectivity between regions does not imply that regions are structurally connected [73], [74]. Given that the significance of functional connectivity within the VAN is unknown, it is important that the connectivity of this network be further studied from an anatomical perspective. It remains an open question whether the lateralized anatomical connections identified here are related to asymmetrical functional connectivity that has been shown in the VAN [10].

### Technical Considerations

Probabilistic tractography is an indirect measure of anatomical connectivity. Inferences can neither be made on directionality of connections nor on the locations of synaptic terminations of identified pathways. However, all the connections we identified are consistent with those previously shown in primates [18], [21]. The number of streamlines calculated between two brain regions, which we have referred to here as “connection strength,” may be influenced by factors unrelated to connectivity such as noise level, modeling errors, and algorithmic errors [16]. Furthermore, when making statistical comparisons using probabilistic tractography, results can be influenced by distance between regions and the size of the regions where tractography is initiated and terminated. We used normalization procedures that account for differences in distance and target size in order to increase the validity of statistical comparisons, but there is no way to accurately account for the effects of distance and target size. Notably, the normalizations have a negligible effect on the main findings of our study (i.e., those based on laterality index) since target distances sizes are similar between the hemispheres.

Probabilistic tractography results can also be influenced by the DWI acquisition scheme, and isotropic voxels are advantageous [75]. In the two acquisitions we used, the voxels were anisotropic with greater size in the z dimension compared to x and y. This anisotropic organization could affect calculation of the probability density function [13], for example resulting in more uncertainty in the z dimension. This could reduce the reliability for detecting superior-inferior pathways. However, since we mainly investigated tracts that traverse most prominently in an anterior-posterior fashion, the effect of anisotropic voxels should be minimal but any effect would be similar in both hemispheres.

Surprisingly there was little improvement in connection strength values comparing 25 and 60 direction DWI acquisition. A previous probabilistic tractography study reported improved sensitivity comparing 12 to 60 directions data within the same subjects to examine major fibre pathways [76]. Tensaouti et al. [77] also reported a significant increase in corticospinal tract volume between 6 and 32 diffusion-encoding direction data, but only a small volume increase between 15 and 32 direction data. In our study, 25 directions seemed to be sufficient for defining tracts between most of the seeds and targets.

### Conclusion

This study demonstrates a novel finding of right-lateralized white matter connectivity between the TPJ and insula, key nodes within the VAN. The results have implications for our understanding of acute and chronic pains and stroke-induced spatial hemineglect.
